# Vascular Endothelial Growth Factor-A Increases the Aqueous Humor Outflow Facility

**DOI:** 10.1371/journal.pone.0161332

**Published:** 2016-09-01

**Authors:** Tomokazu Fujimoto, Toshihiro Inoue, Kei Maki, Miyuki Inoue-Mochita, Hidenobu Tanihara

**Affiliations:** Department of Ophthalmology, Faculty of Life Sciences, Kumamoto University, Kumamoto, Japan; Oregon Health and Science University, UNITED STATES

## Abstract

**Purpose:**

Anti-vascular endothelial growth factor (VEGF) antibody therapy is an effective treatment for ocular angiogenesis. Although the intraocular pressure of some patients increases after anti-VEGF therapy, the effects of VEGF-A on the aqueous humor outflow pathway remain unknown. This study investigated the effects of VEGF-A on the aqueous humor outflow pathway.

**Methods:**

We used human recombinant VEGF121 and VEGF165. Trabecular meshwork (TM) and Schlemm’s canal endothelial (SCE) cells were isolated from the eyes of cynomolgus monkeys. Expression of mRNA coding four VEGF receptors, VEGFR1 (FLT1), VEGFR2 (KDR), neuropilin-1, and neuropilin-2, was examined by RT-PCR. To evaluate the permeability of cell monolayers, we measured transendothelial electrical resistance (TEER). The outflow facility was measured in perfused porcine anterior segment organ cultures treated with 30 ng/mL VEGF121 for 48 h.

**Results:**

Four VEGF-A-related receptor mRNAs were expressed in TM and SCE cells. The TEER of TM cells was not significantly affected by VEGF121 or VEGF165 treatment. In contrast, the TEER of SCE cells was significantly lower 48 h after treatment with 30 ng/mL VEGF121 to 69.4 ± 12.2% of baseline (n = 10), which was a significant difference compared with the control (*P* = 0.0001). VEGF165 (30 ng/mL) decreased the TEER of SCE cells at 48 h after treatment to 72.3 ± 14.1% compared with the baseline (n = 10), which was not a significant difference compared with the control (*P* = 0.0935). Ki8751, a selective VEGFR2 inhibitor, completely suppressed the effect of VEGF121 on SCE cell permeability, although ZM306416, a selective VEGFR1 inhibitor, did not affect the VEGF121-induced decrease in TEER. Perfusion with 30 ng/mL of VEGF121 for 48 h significantly increased the outflow facility compared with the control (47.8 ± 28.5%, n = 5, *P* = 0.013).

**Conclusions:**

These results suggest that VEGF-A may regulate the conventional aqueous outflow of SCE cells through VEGFR2.

## Introduction

Vascular endothelial growth factors (VEGFs) consist of five related growth factors in mammals: VEGF-A, VEGF-B, VEGF-C, VEGF-D, and placental growth factor. VEGFs regulate the physiological functions of vascular and lymphatic vessels. These effects of VEGFs are regulated by three receptor tyrosine kinases including VEGFR1 (FLT1), VEGFR2 (KDR), and VEGFR3 (FLT4), and by co-receptors, such as neuropilins [1]. VEGF-A induces the most potent angiogenic response among the VEGFs, and the effects of VEGF-A are regulated through VEGFR1, VEGFR2, and neuropilins.

Abnormal angiogenesis is associated with several diseases including cancer, inflammatory diseases, and age-related macular degeneration (AMD) [2]. Previous studies have reported that intraocular concentrations of VEGF-A were increased in AMD patients [3]. Recently, anti-VEGF therapies have been commonly used to treat retinal neovascular diseases, such as AMD [4–6]. However, intraocular pressure (IOP) elevation after anti-VEGF treatment has been reported by many clinicians [7–10].

IOP is regulated by the inflow and outflow of aqueous humor in the anterior chamber of the eye. IOP elevation is a risk factor for the development and progression of glaucoma, because sustained IOP elevation causes optic neuropathy [11]. In glaucoma patients, a major cause of IOP elevation is increased aqueous humor outflow resistance through the conventional outflow pathway, which is comprised mainly of the trabecular meshwork (TM) and Schlemm’s canal (SC) [12]. Although abnormal accumulation of extracellular matrix in glaucomatous TM tissue has been hypothesized to lead to increased resistance against aqueous humor outflow [13–15], other causes of resistance related to SC endothelial cells might exist.

Several cytokines, such as monocyte chemoattractant protein-1 (MCP-1) and platelet-derived growth factor (PDGF), have been found in aqueous humor [16–18]. MCP-1 and PDGF have been reported to decrease aqueous humor outflow resistance through TM and SC endothelial (SCE) cells [19, 20]. VEGF has also been detected in aqueous humor [3, 21], although its effects on aqueous outflow resistance were not determined. The purpose of the present study was to investigate the effects of VEGF on the aqueous humor outflow pathway. We examined the barrier function of TM and SCE cells, and the outflow resistance using an anterior segment organ culture perfusion system.

## Materials and Methods

### Materials

Recombinant human VEGF121 and VEGF165 were purchased from Cell Signaling Technology (Danvers, MA, USA). Axitinib, Ki8751, and ZM306416 were purchased from Selleck Chemicals (Houston, TX, USA). The anti-ZO-1 antibody (1:200 dilution) was obtained from Invitrogen (Waltham, MA, USA).

### Cell Culture

Enucleated eyes of cynomolgus monkeys were purchased from Shin Nippon Biomedical Laboratories (Kagoshima, Japan). Primary monkey TM and SCE cells were isolated from the eyes according to a previously described method [22, 23]. Briefly, primary monkey TM and SCE cells were cultured in Dulbecco’s modified Eagle medium (DMEM; WAKO Pure Chemical Industries, Osaka, Japan) in the presence of 10% fetal bovine serum (FBS), glutamine (2 mM), penicillin (100 U/mL), streptomycin (100 μg/mL), and amphotericin B (0.5 μg/mL) at 37°C in 5% CO_2_. Cells were used after between three and five passages.

### Reverse Transcription Polymerase Chain Reaction (RT-PCR)

Total RNA was extracted from cultured TM and SCE cells using NucleoSpin^®^ RNAII (Macherey-Nagel, Düren, Germany). Reverse transcription of the total RNA was performed using Prime Script RT Master Mix (Takara Bio, Shiga, Japan) according to the manufacturer’s protocol. The transcribed cDNA was amplified by the polymerase chain reaction (PCR) (GeneAmp Fast PCR Master Mix, Applied Biosystems, Waltham, MA, USA). The primer sequences were as follows: monkey VEGF receptor (VEGFR)1 (FLT1): forward (F), 5'-GCA AAG CCA CCA ACC AGA AG-3', reverse (R), 5'- AGC CAC ACA GGT GCA TGT TA-3'; monkey VEGFR2 (KDR): (F), 5'- CCC AGA TGA CAA CCA GAC GG-3', (R), 5’-TTG CTG GAC ACC ATT CCA CT-3'; monkey neuropilin-1: (F), 5'-TCT GCC ACT GGG AAC ATG AC-3', (R), 5'-TGC CAT CTC CTG TGT GAT CC-3'; and monkey neuropilin-2: (F), 5'- GGA TCA TCC TGC CCA GCT AC-3', (R), 5'-AGC TGA GAT GGG TTC CAT GC-3'. PCR conditions were 95°C for 10 sec, 35 cycles at 95°C for 1 sec, and 62°C for 15 sec. PCR products were detected by electrophoresis using 2% agarose gels in Tris-acetate-EDTA buffer containing Gel Red^™^ (Nacalai Tesque, Kyoto, Japan).

### Measurement of Monolayer Transendothelial Electrical Resistance (TEER)

TM and SCE cells were cultured to confluence on a Transwell^®^ polyester membrane insert (0.4 μm pore size and 6.5 mm in diameter; Corning, Corning, NY, USA) on 24-well culture plates and serum-starved overnight. TEER was measured using Millicell^®^-ERS (Merck Millipore, Darmstadt, Germany) according to a previously described method [22]. After serum starvation overnight, TM and SCE cells were treated with VEGF121 or VEGF165. TEER was measured at 1, 3, 6, 24, and 48 h after stimulation. To evaluate the effect of VEGF receptor inhibitors, we used 1–10 nM axitinib (VEGFR inhibitor), 3–30 nM Ki8751 (VEGFR2 inhibitor), or 1–10 μM ZM306416 (VEGFR1 inhibitor). TEER was measured at 24 and 48 h after the addition of VEGF inhibitors with VEGF121 or VEGF165. Each experiment was performed at least three times.

### Hoechst 33342 and Propidium Iodide (PI) Dual Staining

To evaluate cell death in TM and SCE cells, we performed the Hoechst 33342 (Dojindo, Kumamoto, Japan) and PI (Invitrogen) double staining method. The cells were grown on 12-well plates at 37°C in 5% CO_2_. After the cells had grown to confluence and were serum-starved overnight, they were treated with VEGF121 or VEGF165 for 48 h. As a positive control, the cells were treated with 1 mM H_2_O_2_ for 2 h and incubated with 1 μg/mL Hoechst 33342 and 1 μg/mL PI for 30 min. The cells were then observed using a fluorescence microscope (IX71; Olympus, Tokyo, Japan), where positive PI staining indicated cell death/damage.

### Anterior Segment Organ Culture Perfusion

Enucleated paired porcine eyes were purchased from Kumamoto Chikusan Ryutsu Center (Kumamoto, Japan). The protocol for the organ culture perfusion has been described previously [22]. Briefly, the eyes were cut at the equator, and the vitreous, lens, iris, and ciliary body were removed from the anterior segment. The anterior segments were placed in custom-designed chambers and perfused with DMEM in the presence of 0.1% FBS, antibiotic, and antimycotic agent at a constant flow (3 μL/min). The anterior segments were placed in a tissue culture incubator at 37°C and 5% CO_2_. After recording the initial baseline for at least 24 h, the perfusion medium was changed to DMEM containing 30 ng/mL VEGF121 and perfused for 48 h. The IOP was recorded on a computer at a rate of 1 Hz. The aqueous humor outflow facility (μL/min/mmHg) was calculated from the perfusion rate (3 μL/min) and the IOP (mmHg).

### Immunocytochemistry

TM and SCE cells were grown to confluence on gelatin-coated glass coverslips, then serum-starved overnight. The cells were treated with 30 ng/mL VEGF121 or VEGF165 for 48 h. The cells were then fixed with 4% paraformaldehyde in phosphate-buffered saline (PBS) for 15 min, permeabilized with 0.5% Triton X-100 in PBS for 12 min at room temperature, and then blocked with serum buffer (10% FBS and 0.2 mg/mL sodium azide in PBS) at 4°C for at least 2 h. After blocking, the cells were treated with the primary antibody overnight at 4°C. The cells were then incubated with anti-rabbit IgG secondary antibody Alexa Fluor 488 (Invitrogen) or Alexa Fluor 546 (Invitrogen) at room temperature for 30 min. To stain F-actin, the cells were incubated with phalloidin-Alexa Fluor 546 (Invitrogen) at room temperature for 30 min. After the cells were mounted with VECTASHIELD^™^ mounting medium with 4',6-diamidino-2-phenylindole (DAPI; Vector Laboratories, Burlingame, CA, USA), fluorescent images of the cells were acquired using an all-in-one epifluorescence microscope (BZ-X710; Keyence, Osaka, Japan). We used a three-filter set for each color observation. The filter- set details were as follows: blue [excitation wavelength (Ex), 360 nm; absorption wavelength (Ab), 460 nm; dichroic mirror wavelength (Di), 400 nm]; green (Ex, 470 nm; Ab, 525 nm; Di; 495 nm); and red (Ex, 545 nm; Ab, 605 nm; Di, 565 nm).

### Statistical Analysis

Data are expressed as means ± standard deviation (SD). Statistical analysis of two groups used Student’s *t*-test or the Wilcoxon rank-sum test. Dunnett’s multiple comparison test or the Tukey–Kramer HSD test was used to compare multiple groups. *P* < 0.05 was considered statistically significant.

## Results

### Expression of VEGF Receptors in TM and SCE cells

**Fig 1:**
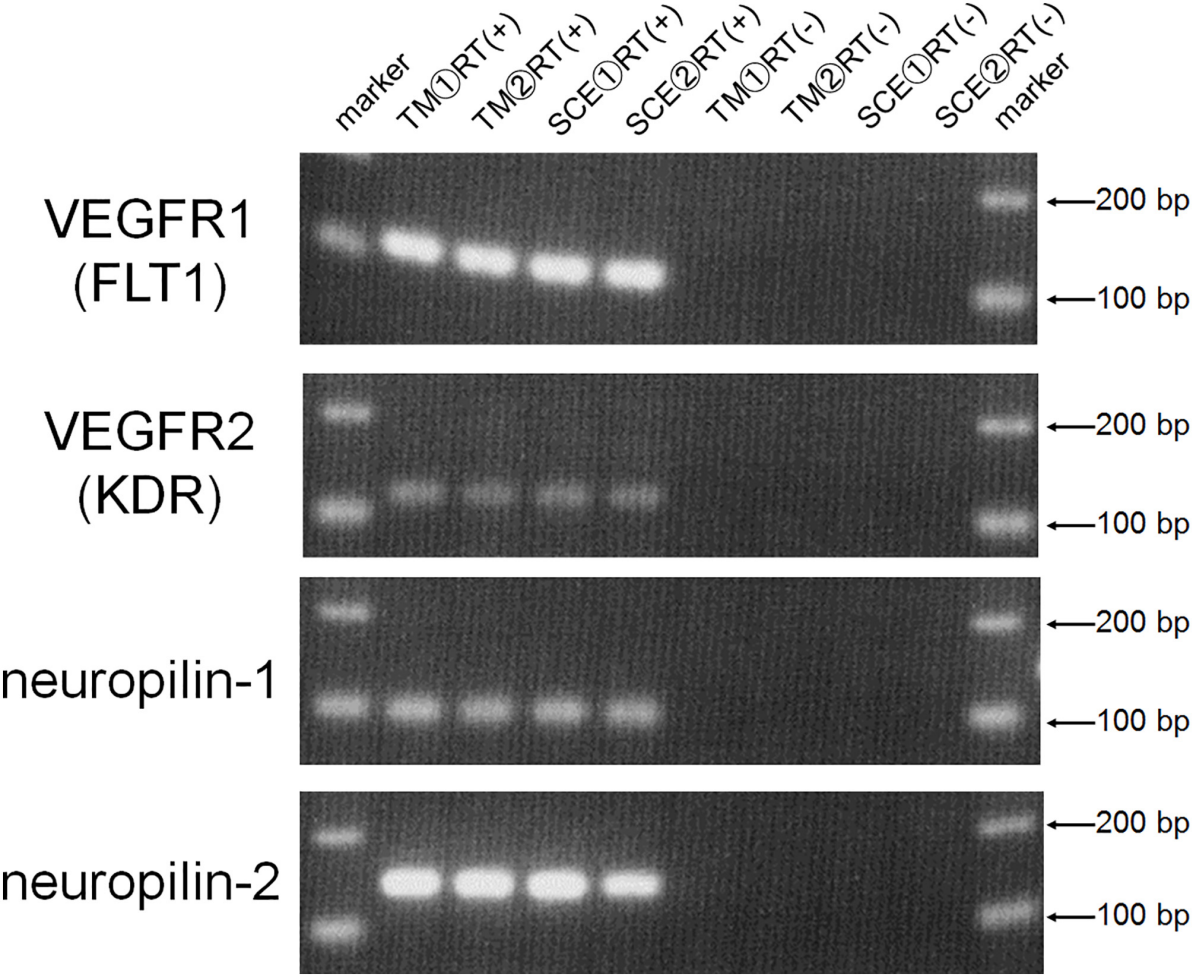
mRNA expression of VEGF receptors in TM and SCE cells. The expression of the mRNAs of four VEGF receptors [VEGFR1 (FLT1), VEGFR2 (KDR), neuropilin-1, and neuropilin-2] in TM and SCE cells was examined by RT-PCR. Samples without reverse transcription [RT(-)] were used as negative controls.

To confirm expression of VEGF-A binding receptors, we performed RT-PCR for VEGFR1, VEGFR2, neuropilin-1, and neuropilin-2 in TM and SCE cells. The expression of all four receptor mRNAs was confirmed in both cells (Fig 1).

### Effect of VEGF on the Barrier Function of TM and SCE Cells

**Fig 2:**
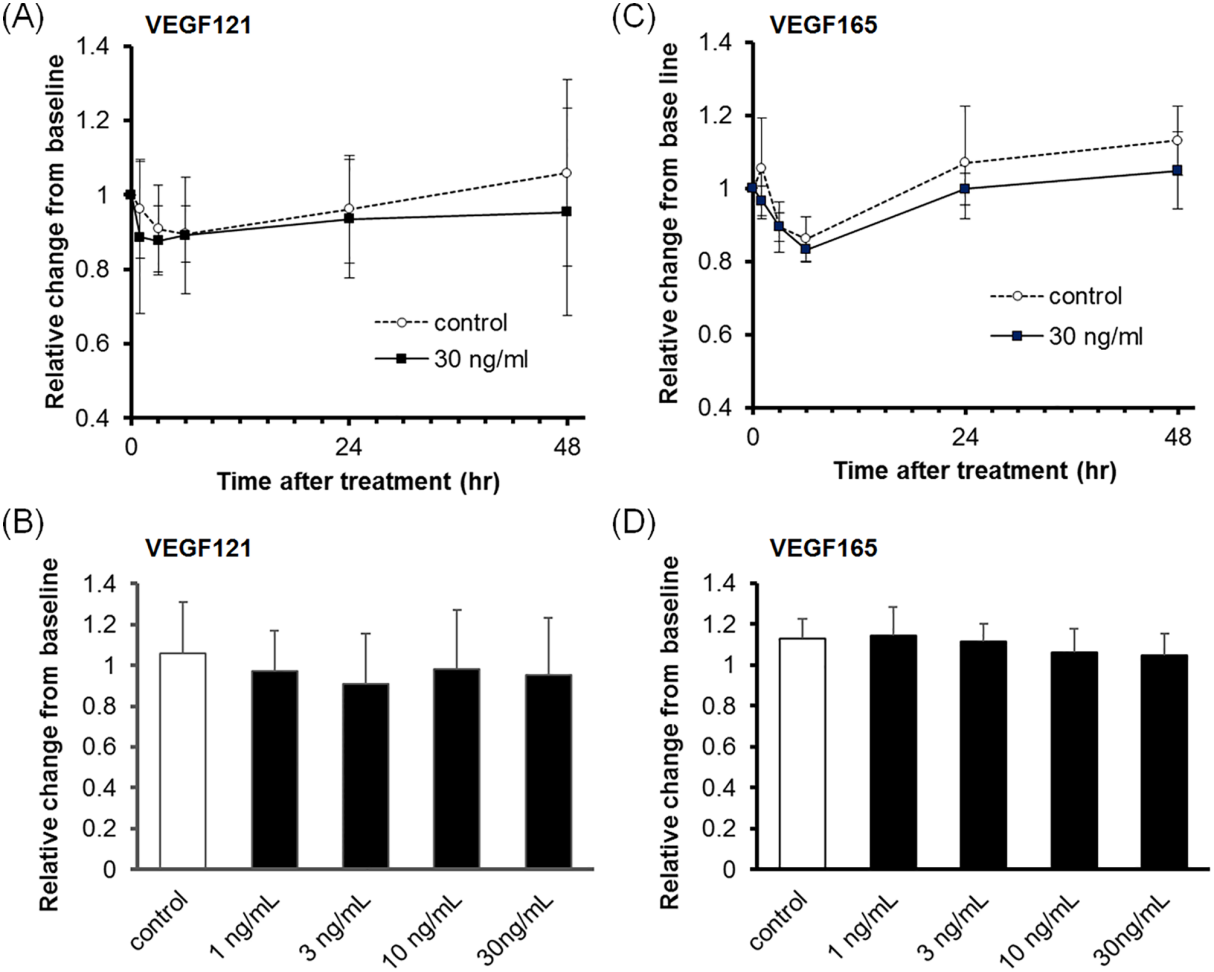
Effect of VEGF121 or VEGF165 on TEER in TM cell monolayers. TM cells were treated with VEGF121 (A, B) or VEGF165 (C, D) for 48 h. (A, C) The time course of TEER changes after 30 ng/mL VEGF121 (A) or VEGF165 (C) treatment. (B, D) TEER changes after VEGF121 (B) or VEGF165 (D) treatment for 48 h. Data are expressed as the mean ± SD from six separate filters (n = 6).

**Fig 3:**
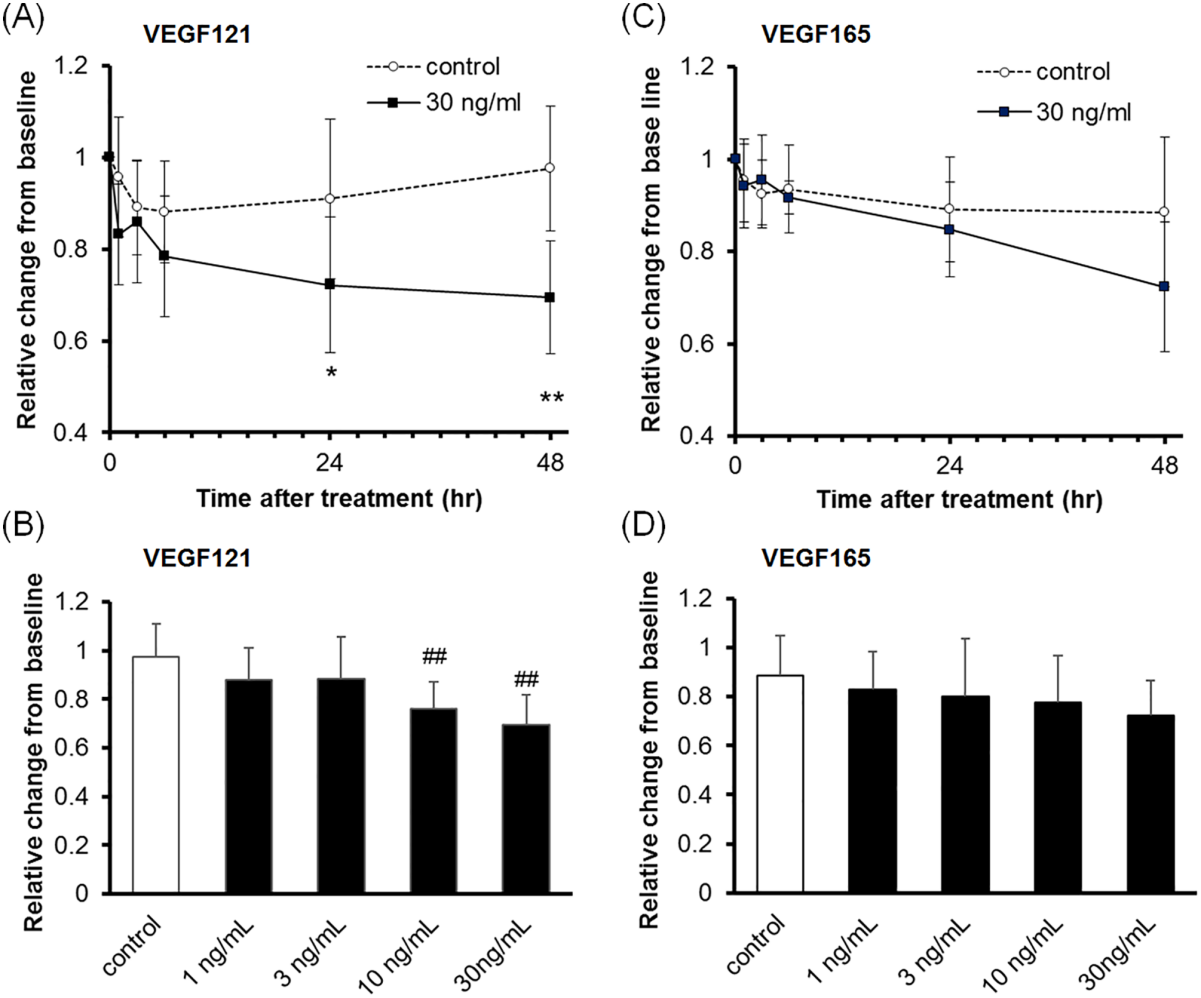
Effect of VEGF121 or VEGF165 on TEER in SCE cell monolayers. SCE cells were treated with VEGF121 (A, B) or VEGF165 (C, D) for 48 h. (A, C) The time course of TEER changes after 30 ng/mL VEGF121 (A) or VEGF165 (C) treatment. (B, D) TEER changes after VEGF121 (B) or VEGF165 (D) treatment for 48 h. Data are expressed as the mean ± SD from ten separate filters (n = 10). *P < 0.05 and **P < 0.01 compared with control using the Wilcoxon rank-sum test. ^#^*P* < 0.05 and ^##^*P* < 0.01 compared with the control using Dunnett’s multiple comparison test.

To evaluate the barrier function of the TM and SCE cell monolayers, we measured TEER. The TEER of TM cells was not affected by VEGF121 or VEGF165 treatment (Fig 2 and Tables 1 and 2). In contrast, the TEER of SCE cells was reduced by VEGF121 treatment in a time- and dose-dependent manner (Fig 3A, 3B and Table 3). Mean (± SD) relative levels of TEER were 75.9 ± 11.5% (n = 10) and 69.4 ± 12.2% (n = 10) compared with the baseline levels at 48 h after 10 ng/mL and 30 ng/mL VEGF121 treatment, respectively. These values were significantly different compared with control values (10 ng/mL, *P* = 0.0033; 30 ng/mL, *P* = 0.0001). VEGF165 (30 ng/mL) lowered the TEER of SCE cells at 48 h after treatment (72.3 ± 14.1% compared to baseline, n = 10), although this was not significantly different compared with control values (Fig 3C, 3D and Table 4).

**Table 1:** The Effect of VEGF121 on TEER in TM Cells.

Group	TEER (ohm·cm^2^), n = 6; mean ± SD	
	Baseline	48 hours
Control	12.82 ± 2.05	13.53 ± 3.97
1 ng/mL VEGF121	14.20 ± 2.74	13.65 ± 3.47
3 ng/mL VEGF121	14.52 ± 3.03	13.15 ± 4.63
10 ng/mL VEGF121	14.37 ± 2.85	13.97 ± 4.85
30 ng/mL VEGF121	14.37 ± 2.68	13.65 ± 5.01

VEGF, vascular endothelial grow factor; TEER, transendothelial electrical resistance; TM, trabecular meshwork; SD, standard deviation.

**Table 2:** The Effect of VEGF165 on TEER in TM Cells.

Group	TEER (ohm·cm^2^), n = 6; mean ± SD	
	Baseline	48 hours
Control	12.33 ± 2.30	13.98 ± 3.17
1 ng/mL VEGF165	12.72 ± 2.32	14.75 ± 4.25
3 ng/mL VEGF165	12.87 ± 2.98	14.40 ± 3.87
10 ng/mL VEGF165	12.98 ± 2.74	13.93 ± 3.96
30 ng/mL VEGF165	13.70 ± 2.71	14.58 ± 4.33

VEGF, vascular endothelial grow factor; TEER, transendothelial electrical resistance; TM, trabecular meshwork, SD, standard deviation.

**Table 3:** The Effect of VEGF121 on TEER in SCE Cells.

Group	TEER (ohm·cm^2^), n = 10; mean ± SD	
	Baseline	48 hours
Control	14.29 ± 3.71	13.72 ± 2.87
1 ng/mL VEGF121	14.26 ± 3.90	12.51 ± 3.57
3 ng/mL VEGF121	14.23 ± 4.03	12.31 ± 3.30
10 ng/mL VEGF121	14.26 ± 4.19	10.88 ± 3.74
30 ng/mL VEGF121	15.12 ± 3.87	10.53 ± 3.34

VEGF, vascular endothelial grow factor; TEER, transendothelial electrical resistance; SCE, Schlemm’s canal endothelial; SD, standard deviation.

**Table 4:** The Effect of VEGF165 on TEER in SCE Cells.

Group	TEER (ohm·cm^2^), n = 10; mean ± SD	
	Baseline	48 hours
Control	15.32 ± 3.79	13.24 ± 2.96
1 ng/mL VEGF165	15.44 ± 3.43	12.71 ± 3.44
3 ng/mL VEGF165	15.22 ± 3.41	12.05 ± 4.45
10 ng/mL VEGF165	15.35 ± 3.93	11.60 ± 3.22
30 ng/mL VEGF165	15.85 ± 3.93	11.46 ± 3.93

VEGF, vascular endothelial grow factor; TEER, transendothelial electrical resistance; SCE, Schlemm’s canal endothelial; SD, standard deviation.

### Effect of VEGF on TM and SCE Cell Death

We investigated cell damage after treatment with VEGF121 or VEGF165 using the PI and Hoechst 33342 double staining method. PI-positive cells were not detected in TM or SCE cells after treatment with 30 ng/mL VEGF121 or VEGF165 for 48 h (data not shown).

### Effect of VEGFR Inhibitors on the Barrier Function of SCE Cells

**Fig 4:**
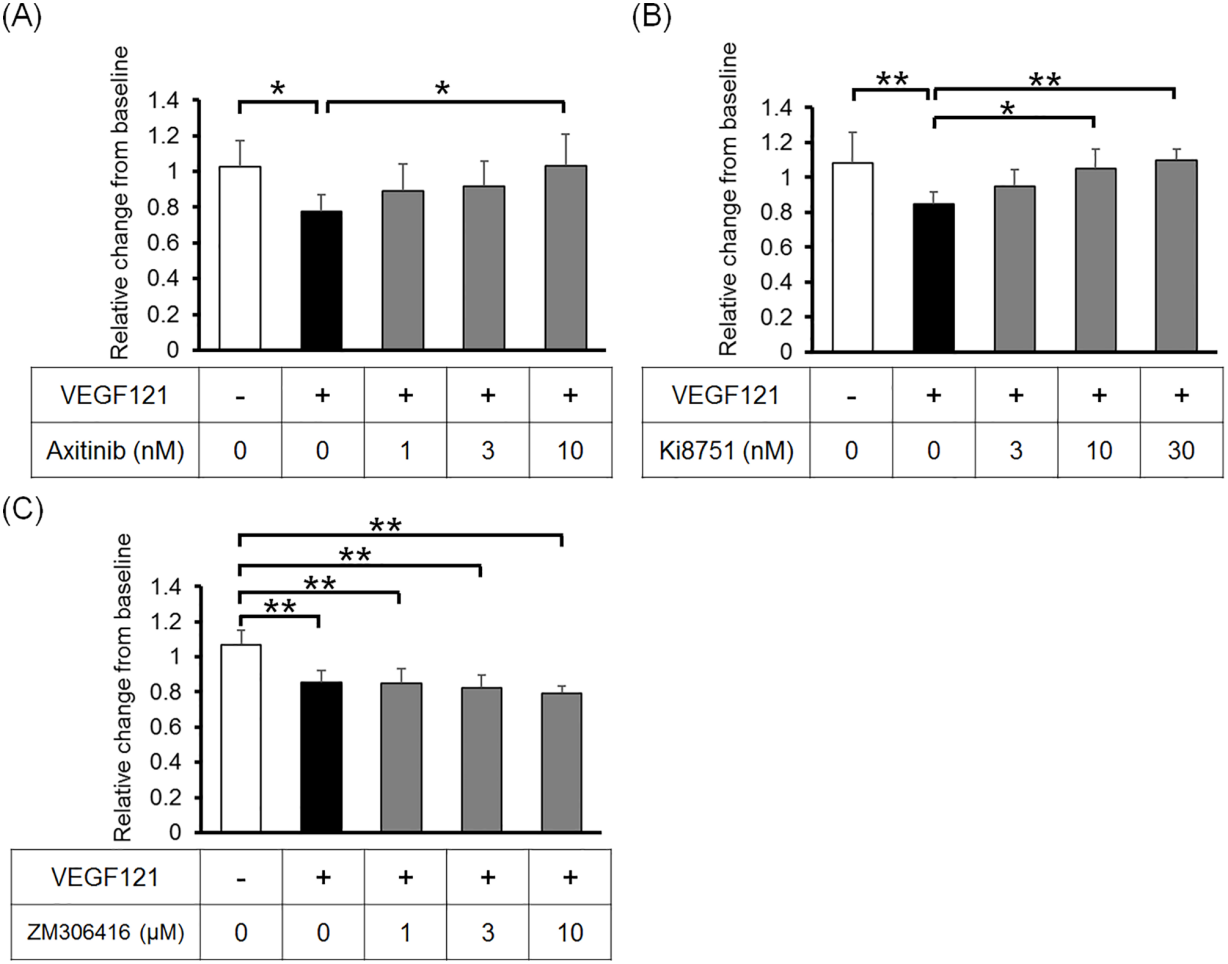
Effect of VEGF receptor inhibitors on TEER in SCE cell monolayers. SCE cells were treated with VEGF121 with or without axitinib (A), Ki8751 (B), or ZM306416 (C) for 48 h. Data are expressed as the mean ± SD from six (A, B) or eight (C) separate filters. *P < 0.05 and **P < 0.01 using the Tukey–Kramer HSD test.

To investigate the effects of VEGFR inhibitors on VEGF-induced permeability in SCE cells, we used three inhibitors: axitinib, Ki8751, and ZM306416. Axitinib, an inhibitor of VEGFR 1, 2, and 3, suppressed the effect of VEGF121 on SCE cell permeability in a dose-dependent manner (Fig 4A). Furthermore, Ki8751, a selective VEGFR2 inhibitor, suppressed the effect of VEGF121 on SCE cell permeability in a dose-dependent manner; the highest dose of Ki8751 completely inhibited the VEGF121-induced permeability (Fig 4B). In contrast, ZM306416, a selective VEGFR1 inhibitor, did not affect the VEGF121-induced permeability of SCE cells (Fig 4C).

### Effect of VEGF on the Outflow Facility of Porcine Eyes

**Fig 5:**
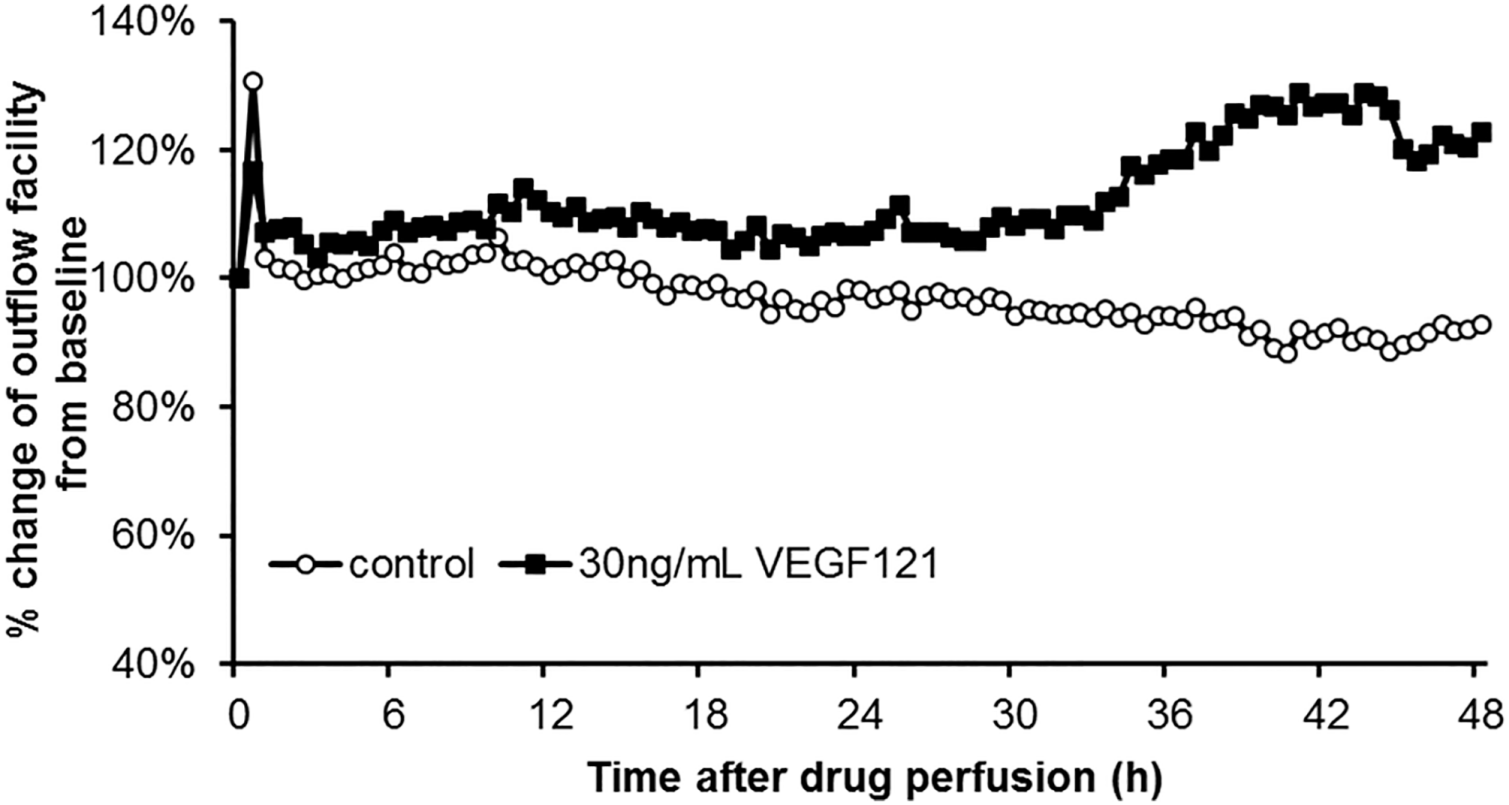
Effect of perfusion with VEGF121 on outflow facility. The anterior segments of porcine eyes were perfused with 30 ng/mL VEGF121 at a constant flow of 3 μL/min at 37°C for 48 h. Data of outflow facility are expressed as the percent change from the baseline value, and expressed as the mean (n = 5).

We evaluated the effect of VEGF on outflow facility using organ culture of anterior ocular segments. The baseline outflow facilities of the control and VEGF121 perfused eyes were 0.517 ± 0.138 and 0.560 ± 0.158 μL/min/mmHg (n = 5, mean ± SD), respectively (Table 5). These baseline outflow facilities were not significantly different. Perfusion with 30 ng/mL VEGF121 for 48 h significantly increased the outflow facility by 47.8 ± 28.5% compared with control values (*P* = 0.0130, n = 5, Fig 5).

**Table 5:** The Effect of VEGF121 on Outflow Facility in Porcine Eyes.

Time	Outflow facility (μL/min/mmHg), n = 5; mean ± SD		*P* value (*t*-test)
	Control	30 ng/mL VEGF121	
Baseline	0.517 ± 0.138	0.560 ± 0.158	0.350
24 hours	0.493 ± 0.095	0.591 ± 0.187	0.194
48 hours	0.458 ± 0.103	0.670 ± 0.151	0.013

VEGF, vascular endothelial growth factor; SD, standard deviation.

### Effect of VEGF on Cell Shape and Actin Structure in TM and SCE Cells

**Fig 6:**
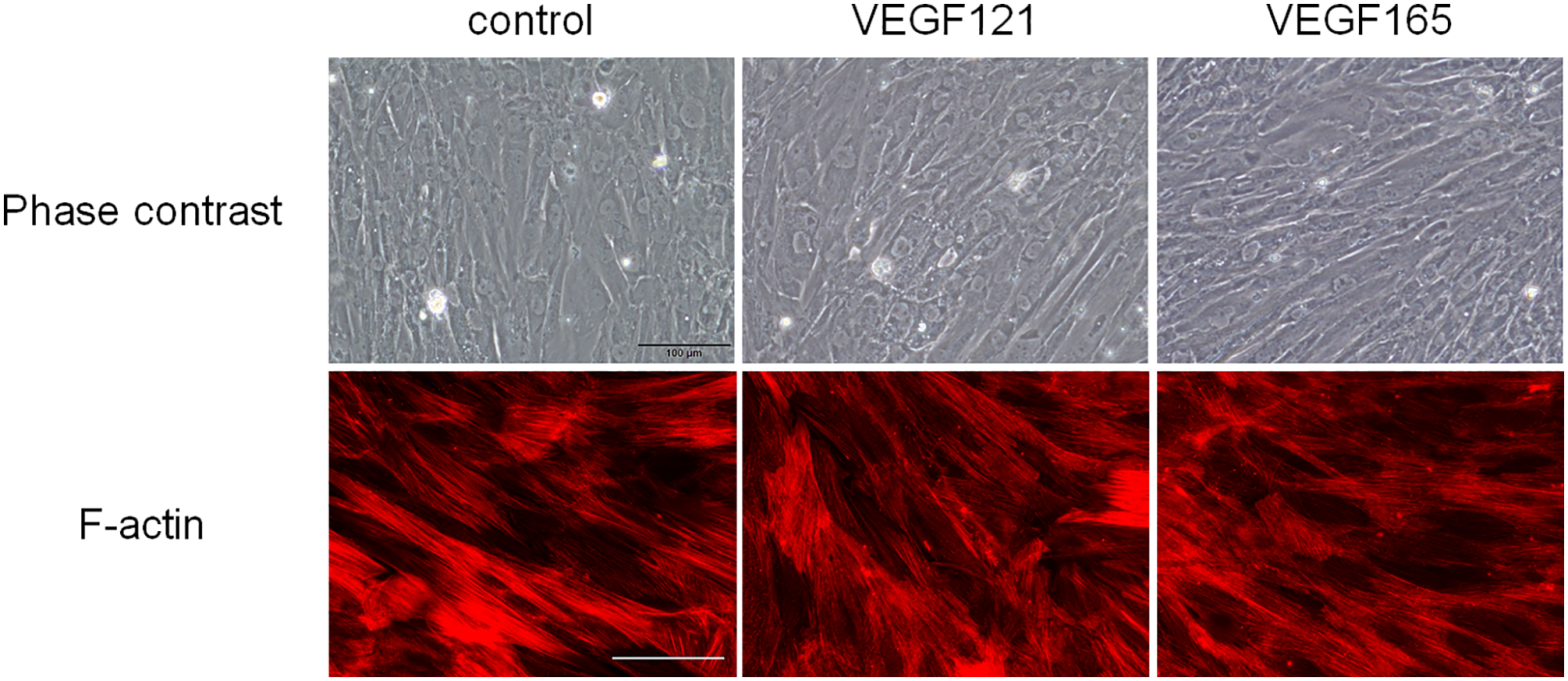
Effect of VEGF121 or VEGF165 on the cell shape and F-actin structure in TM cells. VEGF121 or VEGF165 were used to treat TM cells for 48 h. The top panels show the phase contrast images and the bottom panels show the F-actin-stained images (red). Scale bar, 100 μm.

**Fig 7:**
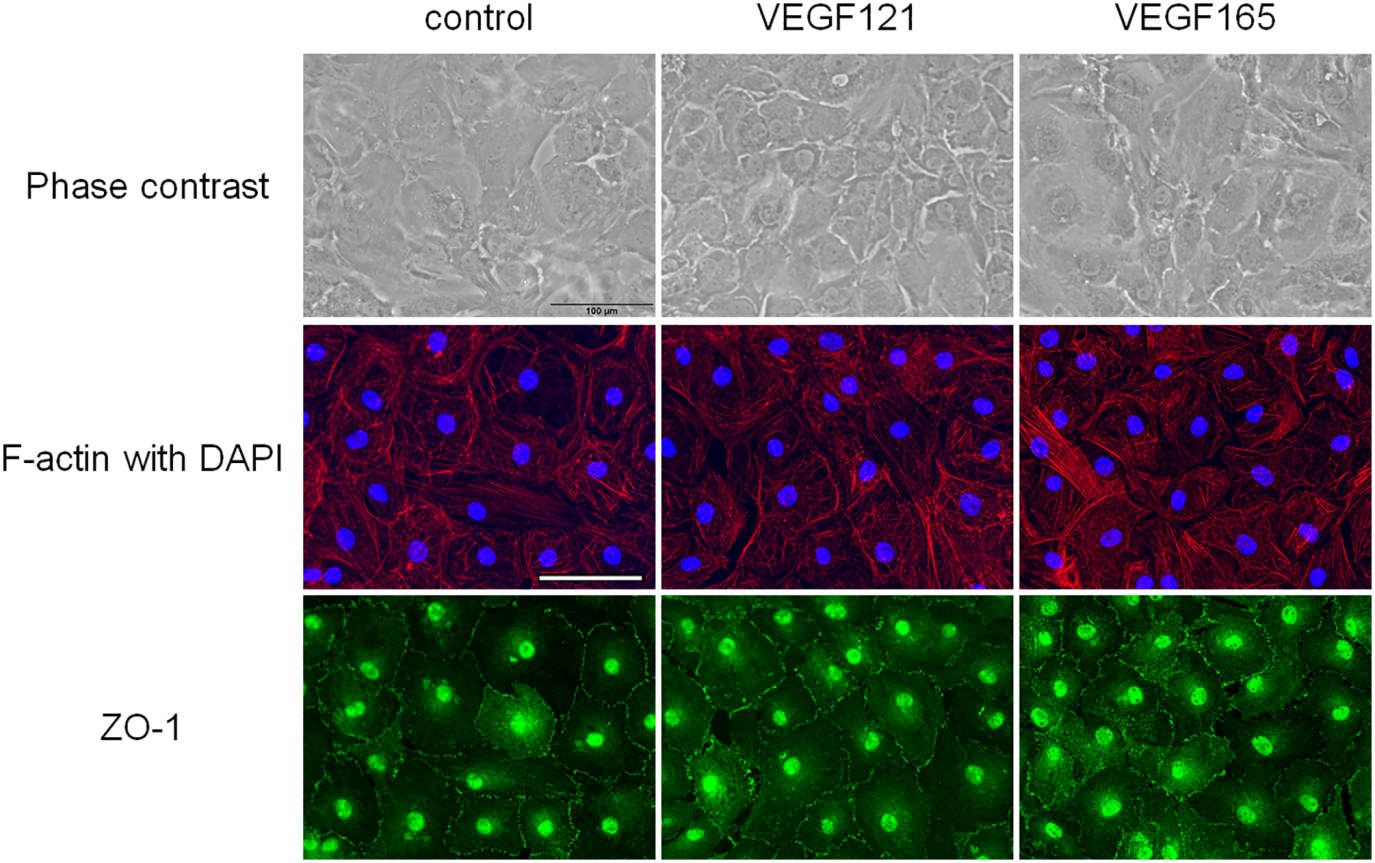
Effect of VEGF121 or VEGF165 on cell shape, F-actin structure, and junctional proteins in SCE cells. VEGF121 or VEGF165 were used to treat SCE cells for 48 h. The top panels show the phase contrast images and the middle panels show the F-actin-stained images (red). Cell nuclei were counterstained with 4',6-diamidino-2-phenylindole (DAPI, blue, middle panel). Bottom panels show a related molecule (ZO-1, green) involved in cell-cell contact. Scale bar, 100 μm.

To investigate the mechanisms related to the VEGF121-induced increase in outflow facilities, we assessed cell shapes and F-actin structures in TM and SCE cells. Following VEGF121 or VEGF165 treatment for 48 h, the morphology and F-actin structures of TM cells and SCE cells showed no obvious changes after VEGF-A treatment (Figs 6 and 7). Furthermore, the immunoreactivity of ZO-1, a junctional protein involved in cell-cell contact, was not affected by VEGF treatment of SCE cells (Fig 7).

## Discussion

Previous studies reported detection of several cytokines in aqueous humor [16–18]. Some of these cytokines were thought to regulate outflow resistance [19, 20]. In the present study, we evaluated the effect of VEGF-A, an aqueous humor cytokine, on aqueous humor outflow. VEGF-A decreased the barrier function of SCE cells and increased outflow facility in porcine anterior segments. Furthermore, the effect of VEGF-A on SCE cell permeability was inhibited by the VEGFR2 specific inhibitor, Ki8751, but not by the VEGFR1 specific inhibitor, ZM306416. These results indicated that VEGF-A regulates outflow resistance through VEGFR2 in SCE cells.

Based on the results of this study, the molecular mechanisms of VEGF-A-induced increase in outflow facility still need to be clarified further. Our previous study reported that MCP-1 decreased the barrier function of SCE cells, probably because MCP-1 changed the intracellular localization of ZO-1 in SCE cells [20]. However, VEGF-A decreased the SCE cell barrier function without changing ZO-1 immunoreactivity after VEGF-A treatment. Furthermore, because cytoskeletal drugs such as Rho kinase inhibitors lower IOP by depolymerizing the actin of TM and SCE cells [23, 24], we evaluated the effects of VEGF-A on actin structure in these cells. The results showed no obvious changes in actin structure. Further research is therefore necessary to identify the mechanisms of the VEGF-A outflow pathway fully.

Two types of IOP elevations after anti-VEGF therapy were reported: transient [25–27] and sustained elevations [7–10]. The cause of transient IOP elevation could be induced by the intravitreal injection itself. However, the causes of sustained IOP elevation after anti-VEGF treatment have not been identified. It could, at least partially, be explained by the side effects of anti-VEGF therapy, because our data indicated that VEGF-A decreased outflow resistance, and the VEGF-A concentration in the aqueous humor was suppressed after anti-VEGF therapy [3, 21].

In conclusion, our results, based on monkey and porcine eyes, show that VEGF121 increased the outflow facility and decreased TEER in SCE cells through VEGFR2, suggesting that VEGF-A regulates aqueous humor outflow through the conventional outflow pathway.
